# A Portable Microfluidic System for Point-of-Care Detection of Multiple Protein Biomarkers

**DOI:** 10.3390/mi12040347

**Published:** 2021-03-24

**Authors:** Nan Li, Minjie Shen, Youchun Xu

**Affiliations:** State Key Laboratory of Membrane Biology, Department of Biomedical Engineering, School of Medicine, Tsinghua University, Beijing 100084, China; lin16@mails.tsinghua.edu.cn (N.L.); smj15@mails.tsinghua.edu.cn (M.S.)

**Keywords:** protein biomarker, microarray, microfluidic cassette, multiplex measurement, immunoassay, point-of-care testing

## Abstract

Protein biomarkers are indicators of many diseases and are commonly used for disease diagnosis and prognosis prediction in the clinic. The urgent need for point-of-care (POC) detection of protein biomarkers has promoted the development of automated and fully sealed immunoassay platforms. In this study, a portable microfluidic system was established for the POC detection of multiple protein biomarkers by combining a protein microarray for a multiplex immunoassay and a microfluidic cassette for reagent storage and liquid manipulation. The entire procedure for the immunoassay was automatically conducted, which included the antibody–antigen reaction, washing and detection. Alpha-fetoprotein (AFP), carcinoembryonic antigen (CEA) and carcinoma antigen 125 (CA125) were simultaneously detected in this system within 40 min with limits of detection of 0.303 ng/mL, 1.870 ng/mL, and 18.617 U/mL, respectively. Five clinical samples were collected and tested, and the results show good correlations compared to those measured by the commercial instrument in the hospital. The immunoassay cassette system can function as a versatile platform for the rapid and sensitive multiplexed detection of biomarkers; therefore, it has great potential for POC diagnostics.

## 1. Introduction

Protein biomarkers are indicators of many diseases, such as cancer, cardiovascular disorders, and infectious diseases, playing a critical role in the early diagnosis and treatment of diseases in clinical practice [1,2,3,4]. However, due to the complex molecular and pathogenic mechanisms of diseases, most cannot be diagnosed by only depending on a single biomarker [5,6,7]. For example, for the accurate diagnosis of cervical cancer at a curable stage, a combination of protein biomarkers is measured, including squamous cell carcinoma antigen (SCC-Ag), serum fragments of cytokeratin (CYFRA), carcinoma embryonic antigen (CEA) and soluble CD44 (sCD44) [8]. The simultaneous determination of a panel of protein biomarkers can significantly improve the specificity and accuracy of the diagnosis. Therefore, the establishment of a multiplexed analytical method with good specificity, sensitivity and speed for the determination of protein biomarkers is one of the most important needs in clinical diagnosis.

For the measurement of protein biomarkers, various promising technologies have been developed, among which the commercialized products mainly include the enzyme-linked immunosorbent assay (ELISA), chemiluminescent or electrochemiluminescent immunoassays, gel electrophoresis and mass spectrometry [9,10,11]. The routine method used for the multiplexed detection of protein biomarkers is ELISA or the ELISA-derived sandwich-type immunoassay in multi-well plates or multiple tubes. Since each well or tube can only be used to detect a single biomarker, multiplexed detection relies on tedious operations or bulky instruments and thus consumes large volumes of samples and reagents and requires a long analytical period. Recently, great efforts have been made to develop alternative methods for multianalyte immunoassays, which involve two strategies: multilabel and spatially resolved assay protocols [12,13]. The multilabel mode utilizes different labels to tag the corresponding analytes for simultaneous detection of various signals from biomarkers [14,15,16]. However, the multilabel strategy is limited by its poor quantification capability and throughput. The spatially resolved mode, which uses a single label, can simultaneously identify all targets at different spatial reaction locations [17,18,19,20]. Many kinds of spatially resolved array-based platforms have been developed in recent years with different signaling patterns, such as fluorescence [21], chemiluminescence [22] and giant magneto resistance [23]. In order to obtain better performances, researchers also explored other approaches, including electrochemical detection [24,25,26,27], surface plasmon resonance [28], cantilever [29], nanowire [30,31], electrical preconcentration [32,33] and various functional materials [34,35]. These approaches usually require microfabrication of the device or complicated material synthesis, which increased the cost of the detection system. Meanwhile, more rigorous operations of immunoassays are needed to ensure a good reproducibility, resulting in sophisticated instrument or intensive labor.

Although the sensitive detection methods and functional materials can improve the performance, the operation of immunoassays usually takes hours and requires highly skilled personnel, which makes it suitable only for non-emergency diagnoses in centralized labs. To overcome these shortcomings, microfluidic technology can be used for the automation and integration of multistep operations by using small cassettes, chips or disks. Several microfluidic platforms [36,37,38,39] have been developed to reduce the detection time and manual operations. On the other hand, protein microarrays have been tested in our previous studies to monitor and quantify multiple targets in human serum and urine [40,41,42]. The versatility and robustness of the microarray system has been demonstrated with thousands of real samples. Therefore, the combination of microarray and microfluidic devices provides a new solution to enhance the performance of multiple-biomarker detection and meet the needs for point-of-care (POC) diagnoses [43,44,45], whereas the majority of the existing microfluidic platforms are still semiautomatic, while fully automated platforms are burdened with large dead volumes and labor-intensive operations. In addition, platforms that rely on bulky off-chip pumps and valves to drive liquid movement result in cumbersome systems and increase the risk of contamination. Recently, the development of ‘self-contained’ microfluidic systems, which integrate all the necessary components for an entire assay, becomes a feasible solution to the aforementioned limitations [46,47]. Dai et al. [48] reported a pump-free microfluidic chip for the detection of multiple biomarkers. The driving of the samples and reagents is conducted by the capillary force along with the gravitational force, which enables the chip to be flux-adaptable and easy-to-use. However, this kind of driving manner cannot actively control the flow and the unidirectional flow also hinders its reaction efficiency. Hu et al. [49] described a self-contained and portable microfluidic chemiluminescence immune sensor for quantitative detection of biomarkers. The microfluidic device contains different reagents in individual reservoirs, which are isolated by on-chip mechanical valves. The reagents are driven by the combination control of a negative pressure and the valves. However, this negative pressure as the only driving force means that this microfluidic chip cannot achieve dynamic reaction, which otherwise needs more reaction time. Sinha et al. [50] developed a field-effect-transistor (FET) sensor array-based microfluidic system for the detection of multiple cardiovascular biomarkers. Although these FET-based biosensors are fast, reliable, compact and extremely sensitive, the fabrication can be complex and costly. Therefore, the demand for a rapid, automated, easy-to-use and portable system for multiplexed immunoassays in POC applications has not been fully satisfied.

In this study, a self-contained microfluidic cassette system is developed for POC detection of multiple protein biomarkers. By combining microarray and microfluidic technology, our system can perform a fully integrated immunoassay for the simultaneous detection of multiple biomarkers in serum samples. The self-contained microfluidic cassette is composed of a Luer syringe, which is used to drive liquid movement, and a rotary valve to regulate or switch the flow direction. The cassette can be divided into two functional parts: the reagent storage module and the immunoassay reaction module. The sample, reagents and waste can be stored in six chambers of the reagent storage module, which increases the practicality of the platform and allows the automation of the immunoassay. The immunoassay reaction module contains a protein microarray and a buffer chamber. Accordingly, a supporting device is constructed to conduct fluidic manipulation in the cassette and record the fluorescence signal. Once the serum sample is loaded into the cassette, multiple steps of the immunoassay are automatically executed, and the entire detection process can be completed within 40 min. Three tumor biomarkers that widely used in clinical diagnosis, alpha-fetoprotein (AFP), CEA and carcinoma antigen 125 (CA125), were chosen to validate the proposed system for the simultaneous detection of multiple biomarkers. Moreover, five clinical samples were tested on our system for further validation.

## 2. Materials and Methods

### 2.1. Design and Fabrication of the Cassette

**Figure 1:**
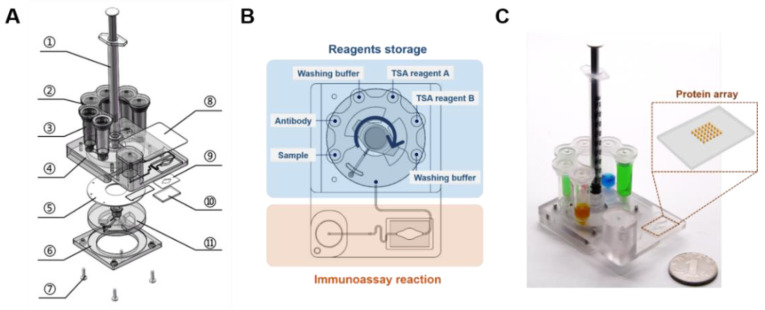
Schematic of the cassette used for the immunoassay. (**A**) The three-dimensional illustration of the cassette in an exploded view, 1. Luer syringe; 2. lid; 3. reagents and waste storage chambers; 4. main body of the cassette; 5. polyurethane washer; 6. cassette base; 7. screws; 8. pressure-sensitive adhesive (PSA) cover slip; 9. double adhesive tape; 10. glass; 11. rotary valve. (**B**) Schematic layout of the microfluidic cassette indicating various features. The blue area represents the reagent storage module, and the yellow area represents the immunoassay reaction module. (**C**) Photo of the cassette. The yellow box shows the glass with a protein microarray.

The proposed microfluidic cassette (5 cm × 6.7 cm × 10.5 cm) designed for the immunoassay is schematically depicted in Figure 1A–C. The center of the cassette contains a Luer syringe (Kangdelai, Shanghai, China) sealed to one outlet of a channel of a polycarbonate (PC) rotary valve via a threaded interface. The other outlet of the channel is used to connect to each liquid chamber (Tiangen Biotech, Beijing, China) inserted in the main body of the cassette. The rotary valve functions as a switching valve inside the cassette to switch between the connection with the Luer syringe in the center and the liquid chambers located outside. When the outlet is precisely aligned to a certain inlet of the liquid chamber, liquid can be transferred between the Luer syringe and the liquid chamber. The polyurethane washer is sandwiched between the main body of the cassette and the rotary valve to avoid liquid leakage. The main body of the cassette and the PC base are tightly fixed in place by screws. Therefore, all of the fluids are guided to the appropriate inlets successively via cooperative control of the self-contained syringe and valve. On one side of the cassette, six chambers that can accommodate up to 1 mL of solution are connected to the main body of the cassette through interfaces. These chambers are covered by a PC lid with venting holes. On the other side of the cassette, a glass layer with a protein microarray printed on its surface is attached to the bottom of the reaction chamber. Double-sided adhesive tape (QL-9970-025, Wuxi Bright Technology, Wuxi, China) is used to bond the chamber and the glass. The reaction chamber is connected with the buffer chamber through a channel (0.3 mm in height and 0.5 mm in width). A pressure-sensitive adhesive (PSA) (90697, Adhesive Research, Beijing, China) cover slip is bonded to the main body of the cassette to seal the chamber and channels. The PC components are machined by a computer numerical control (CNC) machine (Hongyang Laser Co., Ltd., Beijing, China). All of the functional components used for fluid manipulation and reagent storage are integrated in the cassette.

### 2.2. Design of the Supporting Device

**Figure 2:**
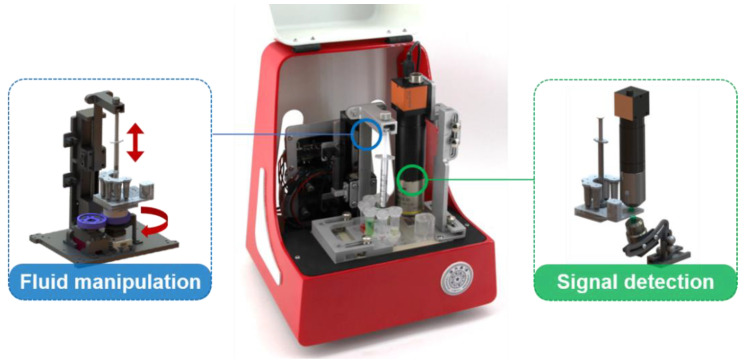
The completed prototype of the supporting device with fluid manipulation and signal detection modules.

As shown in Figure 2, the supporting device (21 cm × 18 cm × 24 cm) is designed for liquid manipulation and fluorescence imaging, which are controlled by an embedded microcontroller unit (MCU) (STM32F103RBT6, STMicroelectronics, Geneva, Switzerland). For liquid manipulation, the syringe piston and the rotary valve are precisely controlled by a stepper motor (28-T6, Shengsida Machinery Equipment Co., Ltd., Suzhou, China) and a digital servo (GDW DS945MG, Shenzhen Huaxiang World Technology Co. Ltd., Shenzhen, China) in a coordinated pattern to achieve basic liquid operations, including liquid transport and mixing. For fluorescence imaging, green light from a light-emitting diode (LED) (530 nm, Quadica Developments, Inc., Alberta, Canada) is filtered by a 510–540 nm bandpass excitation filter (Beijing Bodian Optical Tech. Co., Ltd., Beijing, China) through a microscope objective lens (5× objective lens, Nanjing Jiangnan Novel Optics Co., Ltd., Nanjing, China). The light is used to excite the fluorescent dye on the glass surface after performing the immunoassay. At the same time, the fluorescent images are captured by a digital camera (MVBS30U, CREVIS, Yongin-si, Korea) through a microscope objective lens (MRH00100, Nikon, Shanghai, China) and an optical filter (570 ± 15 nm, Beijing Bodian Optical Tech. Co., Ltd., Beijing, China). The system is powered by external 12 V DC power and communicates with the computer through a USB cable.

### 2.3. Reagents and Materials

All antigens (AFP, CEA, CA125) and their antibodies were purchased from Fapon (Shenzhen, China). Cy3-labelled isotype IgG was purchased from Bioss (Beijing, China). Bovine serum albumin (BSA) and Tween-20 were provided by Sigma. Cy3-labelled tyramide was purchased from ApexBio (Houston, TX, USA). Glass slides were provided by Gous Optics (Shanghai, China) and modified with epoxy groups by CapitalBio (Beijing, China). The printing buffer was also purchased from CapitalBio. PBS (10 mM, pH = 7.4) was provided by Solarbio (Beijing, China). PBST (PBS with 0.1% Tween-20) was prepared as the washing buffer. The water used in the experiments was purified with a Milli-Q system (Millipore, Beijing, China).

### 2.4. Printing and Immobilization of the Antibodies

The antibodies were diluted in the printing buffer and then spotted onto epoxy-group-modified glass slides using a printing robot (Personal Arrayer 16, CapitalBio). After spotting, the glass slide was kept at room temperature for 8 h. The epoxy groups on the slides reacted with the free amino groups of the antibodies, covalently linking the antibodies to the glass surface. After that, the glass slide was blocked in 3% BSA solution for 1 h. The glass slides were washed 3 times and kept at 4 °C before use. There were two types of contact-printed arrays on the glass slide. The first one was used for optimizing the spotting concentrations (Figure S1A), and the second one was used to determine the spotting concentrations of antibodies for detection (Figure S1B).

### 2.5. Optimization of the Antibody Concentrations

The dosage of the capture antibody immobilized on the glass slide and the horseradish peroxidase (HRP)-labelled antibody were both optimized for each biomarker. For example, different concentrations of the capture antibody for AFP were spotted onto the glass slide, and different dilution ratios of HRP-labelled antibody were utilized to measure the AFP simulated solution (10 ng/mL). The optimal conditions were chosen according to the concentration, yielding a strong detection signal and low reagent consumption.

### 2.6. Immunoassays

The sample, reagents and buffer were loaded into the cassette. The immunoassay was conducted with the supporting device according to the programmed procedure. Simulated samples of AFP, CEA and CA125 at a series of concentrations were measured with our system. Calibration curves were established according to the results. Similarly, clinical serum samples were also tested on our system.

### 2.7. Data Acquisition and Analysis

Once the on-cassette immunoassay was completed, the fluorescent signal of the detection microarray was excited by an LED and captured by the camera. Differences in the median fluorescence intensity (MFI) between the signal and the blank were determined by LuxScan 3.0 software (CapitalBio). According to the data extracted from the images, standard curves for the three biomarkers were plotted, and the concentration of each target in the clinical samples was accurately quantified with the corresponding curves.

## 3. Results

### 3.1. Workflow of the Immunoassay Cassette

**Figure 3:**
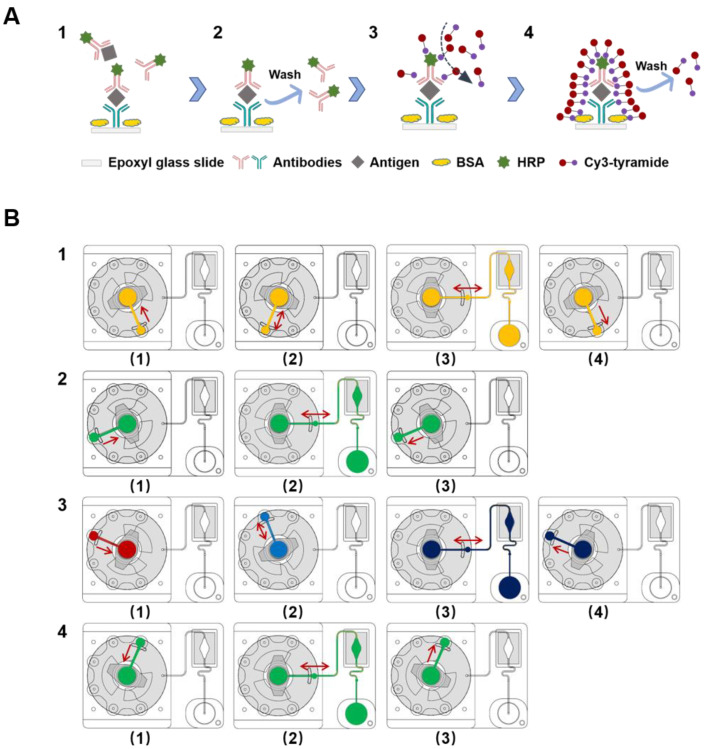
The cassette processing protocol. (**A**) Schematic showing the process of the immunoassay. (**1**) Antibody–antigen reaction; (**2**) washing; (**3**) horseradish peroxidase (HRP)-catalyzed signal amplification; (**4**) washing. The Cy3-tyramide molecule can be catalyzed by HRP under a H_2_O_2_ environment, and the activated tyramide can covalently bind to certain amino acid residues of the proximate proteins. (**B**) Illustration of the entire flow control system of the cassette: (**1**) the 200 μL serum sample is mixed with lyophilized antibody and then placed into the reaction chamber; (**2**) after completing the antibody–antigen reaction, the glass is washed with 800 μL washing buffer; (**3**) 200 μL TSA reagent A and 200 μL TSA reagent B are mixed prior to transfer into the reaction chamber; (**4**) the glass is washed with 800 μL washing buffer.

The cassette proposed in this study is designed to accomplish the simultaneous detection of multiple biomarkers in a “sample-in to answer-out” manner. The process of the immunoassay and the control program of the cassette are described below (Figure 3). A video is supplied to visualize this process (Supplementary Movie S1). Before the assay, the channel and reaction chamber of the cassette were blocked with PBST containing 2% BSA for 1 h to reduce nonspecific binding. The corresponding reagents for the immunoassay were preloaded into the storage chambers of the cassette. After adding the serum sample (200 μL), the cassette was fixed to the supporting device. The automated manipulation procedure for the immunoassay was executed as follows:

(1) Antibody–antigen reaction. The rotary valve was rotated to engage the inlet of the sample chamber. The serum sample (200 μL) was driven into the Luer syringe, and then the rotary valve was rotated clockwise to connect the inlet of the antibody chamber. The serum sample was dispensed into the antibody chamber and completely mixed with the lyophilized antibody for 1 min at a flow rate of 50 μL/s. After dissolving, the mixture was aspirated back into the Luer syringe, and the rotary valve was rotated clockwise to connect the reaction chamber. Subsequently, the mixture was transferred into the reaction chamber and gently pushed and pulled back and forth by the syringe to accelerate the reaction for 18 min at a flow velocity of 10 μL/s. After the affinity binding reaction, the waste was transferred back to the sample chamber (Figure 3B1). The total time required for the antibody–antigen reaction step was 20 min.

(2) Washing. After the affinity binding step, the rotary valve was switched to connect the inlet of the first washing buffer chamber, and the buffer was driven into the Luer syringe. Then, the rotary valve was rotated clockwise to connect the reaction chamber again, and the reaction chamber was washed by pushing and pulling the washing buffer back and forth for 5 min (800 μL at 20 μL/s). After the washing process, the waste was discarded into the first washing chamber (Figure 3B2).

(3) HRP-catalyzed signal detection. Following the first washing process, the rotary valve was switched to connect the Luer syringe and the inlet of the tyramide signal amplification (TSA) reagent A chamber. After 200 μL TSA reagent A was driven into the Luer syringe, the rotary valve was rotated clockwise to connect to the TSA reagent B chamber. Then, TSA reagent A was transferred into the TSA reagent B chamber and mixed with 200 μL TSA reagent B for 30 s at a 50 μL/s flow velocity. Afterwards, the 400 μL volume of TSA reagent mixture was transferred into the reaction chamber to detect the signal from the protein microarray for 9 min at a 10 μL/s flow velocity. After the signaling reaction, the waste was discarded into the TSA reagent A chamber (Figure 3B3).

(4) Washing. The second washing step followed a process similar to that of the first washing step (Figure 3B4).

It is noticed that bubble trapping issues may sometimes occur in the reaction chamber. Though it has finite effect on the detection result as the bubbles are apt to attach to the top PSA in the reaction chamber, further upgrade of the cassette may eliminate these issues. Long and narrow chambers with rounded corners are preferred. In addition, injection molding of the cassette and hydrophilic modification on the surface of channels and chambers can also help to reduce the bubble formation.

The entire immunoassay process can be accomplished within 40 min. The TSA reaction steps were applied in this system to improve the sensitivity of the immunoassay [50]. Moreover, the above workflow can be easily reprogrammed to perform other experimental protocols.

### 3.2. Optimization of the Concentrations of Antigen and Antibody

First, the concentration of the capture antibody used for immobilization by printing on the glass slide along with the concentration of the HRP-labelled antibody were optimized to improve the performance of the immunoassay. As shown in Figure S2, a comparatively better signal was generated at a concentration of 0.6 mg/mL for the immobilization of the AFP capture antibody and at the dilution of 1:250 for the HRP-labelled AFP antibody. For CEA and CA125, the antibody concentrations used for printing were 0.6 and 0.4 mg/mL, respectively. The preferred dilution ratios of the HRP-labelled antibody for these three biomarkers were all 1:250. Second, according to the optimized results, the HRP-labelled antibodies for the three biomarkers were mixed and lyophilized in a tube in the cassette. During the immunoassay, the antibodies were dissolved for the automated detection of simulated and clinical samples.

### 3.3. Determination of the Specificity

Multitarget detection in a single chamber can result in cross-reactions between different antigens and antibodies during the immunoassay. Therefore, the specificity of the immunoassay was determined by evaluating the cross-reactivity (CR) of the reactions. For example, the CR between AFP and CEA-Ab was calculated by using Equation (1):

CR (%) = MFI_(AFP, CEA-Ab)_/MFI_(AFP, AFP-Ab)_ × 100% (1)

**Figure 4:**
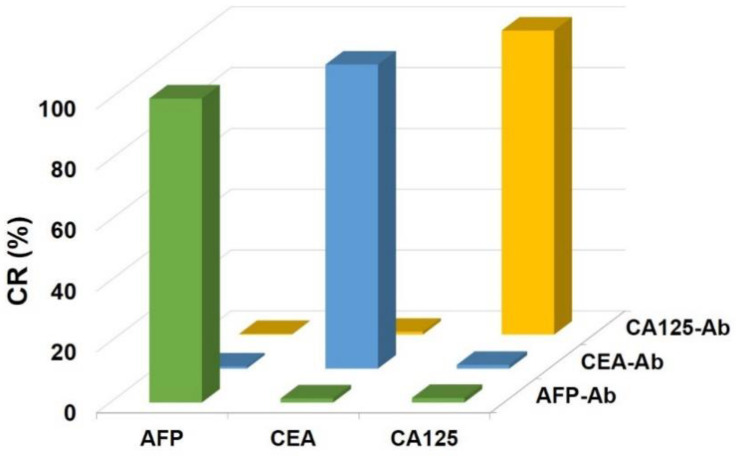
Cross-reactivities of the three pairs of antigens and antibodies.

The CR values were obtained by detecting three simulated samples containing only one target. The concentrations of the AFP, CEA and CA125 samples were 8 ng/mL, 8 ng/mL, and 32 U/mL, respectively. As shown in Figure 4, the values of the CR between unmatched antigens and antibodies were all below 2%, suggesting a high specificity of the binding between the antigens and their respective antibodies.

### 3.4. Calibration Curves of the Three Biomarkers

**Figure 5:**
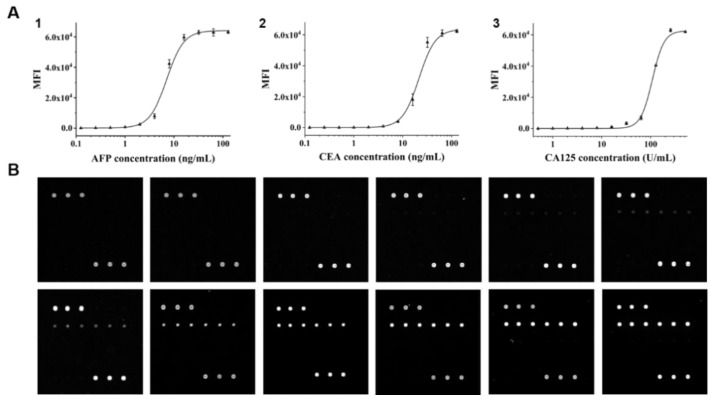
Establishment of the standard curves. (**A**) Calibration curves for the immunoassays of tumor markers. (**1**) Standard curve for alpha-fetoprotein (AFP); (**2**) standard curve for carcinoembryonic antigen (CEA); (**3**) standard curve for carcinoma antigen 125 (CA125). The error bars show the standard deviations of triplicate measurements. (**B**) Representative fluorescence images of microarrays during detection of AFP with simulated standard samples. The concentrations of AFP were 0, 0.125, 0.25, 0.5, 1, 2, 4, 8, 16, 32, 64 and 128 ng/mL (from left to right and top to bottom).

Simulated samples with a series of different concentrations for each biomarker were prepared and analyzed by following the operation protocol to obtain the standard curves (Figure 5). The fitting equations and the limits of detection (LODs) are listed in Table 1. The LOD values were conservatively calculated by using the average MFI value of the blank sample plus three times the standard deviation, and the concentrations for AFP, CEA and CA125 were calculated to be 0.303 ng/mL, 1.870 ng/mL, and 18.617 U/mL, respectively. These concentrations are all within the normal range of those in a healthy individual. Therefore, our system is capable of disease monitoring and diagnosis. However, the Cy3-tyramide signaling method we applied in the immunoassay resulted in a smaller linear range than ELISA (0.01 to 1 ng/mL) [51], indicating that further optimization is needed in the future. In addition, the linear range could also be shifted according to clinical requirements by altering the concentration of the printing antibody and HRP-labelled antibody, the reaction time and other signal amplification parameters.

### 3.5. Detection of Clinical Samples

Clinical samples were also collected to further validate the use of the cassette system for diagnosis via protein biomarker detection. As shown in Table 2, the relative deviations of the concentrations of AFP, CEA, and CA125 in five clinical samples measured by our system and in the hospital were all within 20%, suggesting the potential of our system for clinical use. The accuracy of our system can be affected by the machining of the cassette compartments, the manual assembly of the cassette, and the signal extracted from the immunoassay array. Therefore, the accuracy of our system still has the potential for further improvement. In fact, there is still a gap between our system and a ready-to-use product in clinical applications. Additionally, we are still dedicated in the improvement of our system and hope for a better performance in the follow-up study.

These results clearly demonstrated that multitarget detection of protein biomarkers can be achieved with our system. Fully automated detection of three protein biomarkers in clinical samples can be completed within 40 min in a “sample-in and answer-out” manner. In addition, our system is capable of functioning as a platform for the detection of more than three biomarkers according to the microarray-based immunoassay. Several previous methods for multi-target detections have been listed in Table S1. It is found that when combining immunoassay with microfluidic devices, they are apt to have several compromises in their sensitivity, simplicity, speed, and cost. Our method has comprehensive advantages regarding self-containment, detection time, sensitivity, integration and fabrication complexity. These features make our system suitable for POC applications.

## 4. Conclusions

In this study, a self-contained multiplexed immunoassay cassette system was developed by integrating a protein microarray into a microfluidic cassette for automated immunoassay. The entire process, including the antibody–antigen reaction, first wash, signal amplification and second wash, can be automatically accomplished within 40 min. The system is composed of a disposable cassette and a handheld device. There is no exchange of liquids between the cassette and the device, thus minimizing the possibility of carry-over contamination. The cassette system was applied to simultaneously detect AFP, CEA and CA125 in human serum, and good sensitivity and repeatability were obtained. Moreover, five clinical samples were detected with this system, and the obtained results were in acceptable agreement with those measured by the commercial instrument in the hospital. These results demonstrate that the presented immunoassay cassette system was sensitive, easy to use, rapid and could be easily expanded for the simultaneous detection of various kinds of protein biomarkers for POC applications.

## Figures and Tables

**Table 1:** Standard curves and the limits of detection (LODs).

Target	Standard Curve	Adj. R^2^	LOD
AFP	y_AFP_ = 64,114.05 − 63,980.01/[1 + (x/7.10)^2.4908^]	0.9940	0.303 ng/mL
CEA	y_CEA_ = 63,554.89 − 63,508.52/[1 + (x/21.50)^2.5841^]	0.9935	1.870 ng/mL
CA125	y_CA125_ = 62,549.65 − 62,485.99/[1 + (x/108.58)^3.6293^]	0.9979	18.617 U/mL

**Table 2:** Detection of five clinical samples.

No.	Measured in Hospital			Measured in Cassette System					
	AFP (ng/mL)	CEA (ng/mL)	CA125 (U/mL)	AFP (ng/mL)	RD ^1^ (%)	CEA (ng/mL)	RD (%)	CA125 (U/mL)	RD (%)
1	2.75	2.39	7.51	2.66	3.4	2.62	9.5	7.82	4.1
2	3.57	2.43	27.9	3.39	5.0	2.58	6.3	30.89	10.7
3	1.63	2.52	8.65	1.64	0.5	2.24	11.0	10.02	15.8
4	6.08	1.97	19.14	6.49	6.7	2.08	5.4	15.52	18.9
5	8.59	2.14	12.2	7.52	12.5	2.30	7.5	11.91	2.4

^1^ RD stands for the relative deviation between the concentration measured by the cassette system and that measured in hospital (by Roche cobas e601). RD (%) = |C_(Hospital)_ − C_(Cassette system)_|/C_(Hospital)_ × 100%.

## Supplementary Materials

The following are available online at https://www.mdpi.com/2072-666X/12/4/347/s1, Figure S1: (A) Printing design of the capture antibody on the optimization glass. Antibodies of AFP, Antibodies against AFP, CEA, and CA125 at different concentrations in printing buffer were printed on the surface of the epoxy group-modified glass. (B) The design for printing on the detection glass consisted of the optimal concentration of each antibody and controls (Cy3-IgG and printing buffer), Figure S2: Optimization of the antibody concentrations. (A) AFP, (B) CEA, (C) CA125, title, Table S1. Summary of POC multi-target immunoanalyzers, Movie S1. Automated processing with the cassette system. The video shows the on-cassette fluid control steps, and the sample and reagents are represented by different colored dyes.
