# Supplementary material for: A Portable Microfluidic System for Point-of-Care Detection of Multiple Protein Biomarkers
Source: Micromachines (Basel). 2021 Mar 24;12(4):347. doi: 10.3390/mi12040347 (PMC8063924; doi:10.3390/mi12040347)
Supplement: Supplementary file 1 [file micromachines-12-00347-s001.zip › micromachines-1158545-supplementary/micromachines-1158545-Supplementary.pdf]

micromachines

MDPI

Article

A Portable Microfluidic System for Point-of-Care Detection of Multiple Protein Biomarkers

Nan Li †, Minjie Shen † and Youchun Xu *

State Key Laboratory of Membrane Biology, Department of Biomedical Engineering, School of Medicine, Tsinghua University, Beijing 100084, China; lin16@mails.tsinghua.edu.cn (N.L.); smj15@mails.tsinghua.edu.cn (M.S.)

* Correspondence: xyc2012@mail.tsinghua.edu.cn; Tel.: +86-10-62796071

† These authors contributed to this work equally.

A

Ab-C_1	Ab-C_1	Ab-C_1	Ab-C_1	Ab-C_1	Ab-C_1	Ab-C_1
Ab-C_2	Ab-C_2	Ab-C_2	Ab-C_2	Ab-C_2	Ab-C_2	Ab-C_2
Ab-C_3	Ab-C_3	Ab-C_3	Ab-C_3	Ab-C_3	Ab-C_3	Ab-C_3
Ab-C_4	Ab-C_4	Ab-C_4	Ab-C_4	Ab-C_4	Ab-C_4	Ab-C_4
Ab-C_5	Ab-C_5	Ab-C_5	Ab-C_5	Ab-C_5	Ab-C_5	Ab-C_5
Ab-C_6	Ab-C_6	Ab-C_6	Ab-C_6	Ab-C_6	Ab-C_6	Ab-C_6
Ab-C_7	Ab-C_7	Ab-C_7	Ab-C_7	Ab-C_7	Ab-C_7	Ab-C_7

B

Cy3-IgG	Cy3-IgG	Cy3-IgG	Printing buffer	Printing buffer	Printing buffer
AFP-Ab	AFP-Ab	AFP-Ab	AFP-Ab	AFP-Ab	AFP-Ab
CEA-Ab	CEA-Ab	CEA-Ab	CEA-Ab	CEA-Ab	CEA-Ab
CA125-Ab	CA125-Ab	CA125-Ab	CA125-Ab	CA125-Ab	CA125-Ab
Printing buffer	Printing buffer	Printing buffer	Cy3-IgG	Cy3-IgG	Cy3-IgG

Figure S1. (**A**) Printing design of the capture antibody on the optimization glass. Antibodies of AFP, Antibodies against AFP, CEA, and CA125 at different concentrations in printing buffer were printed on the surface of the epoxy group-modified glass. (**B**) The design for printing on the detection glass consisted of the optimal concentration of each antibody and controls (Cy3-IgG and printing buffer).

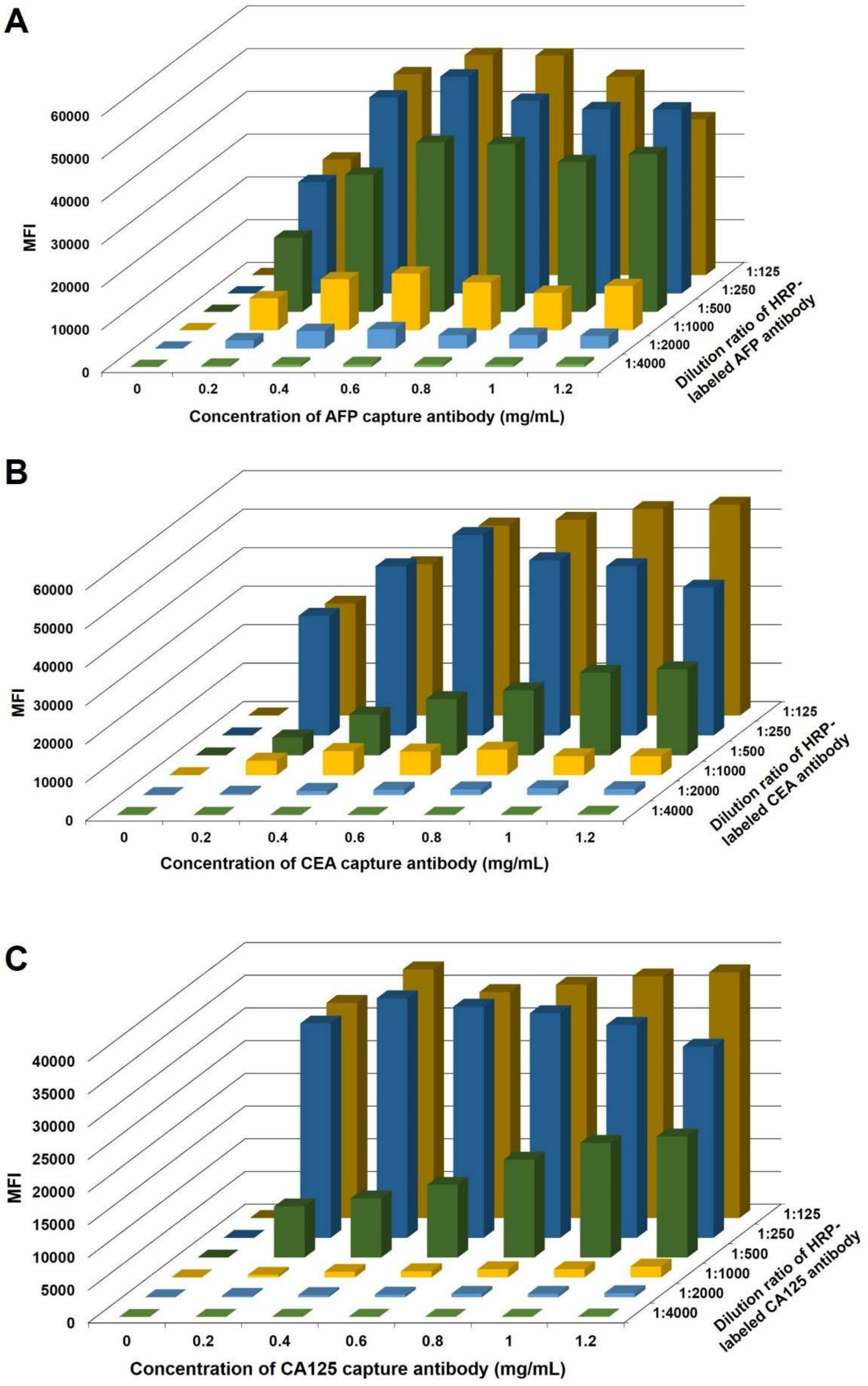

Figure S2. Optimization of the antibody concentrations. (A) AFP, (B) CEA, (C) CA125.

Table S1. Summary of POC multi-target immunoanalyzers [1–3].

.	Method	Microfluidic platform	Self-contained	Time	LOD	Sample to answer	Fabrication of disposables
Noroozi et al. [4]	ELISA and microarray chip	Semi-automated centrifugal disc	No	>1 h	_	No	Simple
Kloth et al. [5]	Chemiluminescence and microarray chip	Microarray chip connected with pump and valve	No	6 min	Penicillin G 1.1 µg/L	Yes	Simple
Chen et al. [6]	Fluorescence and microarray chip	Microarray chip connected with pump and valve	No	30 min	T2 0.12 ng/mL, OTA 1.24 ng/mL	Yes	Simple
Luo et al. [7]	Chemiluminescence and microarray chip	Automatic biochip reader	No	1 h	AFP 2 ng/mL, CEA 0.5 ng/mL, CA125 2.3 U/mL	Yes	Simple
Lee et al. [8]	Microbead-based suspension ELISA assays	Centrifugal disc	Yes	30 min	HBsAg 0.51 ng/mL	Yes	Moderate
Chikkaveeraiah et al. [9]	Electrochemical immunosensor array	Array chip connected with pump and injector	No	1.15 h	PSA 0.23 pg/mL, IL-6 0.30 pg/mL	Yes	Moderate
Balakrishnan et al. [10]	Multi-electrode poly-silicon nanogap	Chip connected with pump and valve	No	1 h	APTES 0.56 mIU/mL, GPMS 2.93 mIU/mL	No	Moderate
Gao et al. [11]	Giant magneto resistance (GMR) multi-biomarker immunoassay biosensor	Test card	Yes	15 min	AFP 0.52 ng/mL, CEA 0.27 ng/mL	Yes	Complex
This work	Florescence and microarray chip	Cassette	Yes	40 min	AFP 0.303 ng/mL, CEA 1.870 ng/mL, and CA125 18.617 U/mL	Yes	Simple

References

1. Lei, R.; Huo, R.; Mohan, C. Current and emerging trends in point-of-care urinalysis tests. *Expert Rev Mol Diagn* **2020**, *20*, 69–84.
2. Poschenrieder, A.; Thaler, M.; Junker, R.; Luppa, P.B. Recent advances in immunodiagnostics based on biosensor technologies-from central laboratory to the point of care. *Anal Bioanal Chem* **2019**, *411*, 7607–7621.
3. Lode, P. Point-of-care immunotesting: Approaching the analytical performance of central laboratory methods. *Clin Biochem* **2005**, *38*, 591–606.
4. Noroozi, Z.; Kido, H.; Peytavi, R.; Nakajima-Sasaki, R.; Jasinskas, A.; Micic, M.; Felgner, P.L.; Madou, M.J. A multiplexed immunoassay system based upon reciprocating centrifugal microfluidics. *Rev Sci Instrum* **2011**, *82*, 064303.
5. Kloth, K.; Niessner, R.; Seidel, M. Development of an open stand-alone platform for regenerable automated microarrays. *Biosens Bioelectron* **2009**, *24*, 2106–2112.
6. Chen, Y.; Meng, X.; Zhu, Y.; Shen, M.; Lu, Y.; Cheng, J.; Xu, Y. Rapid detection of four mycotoxins in corn using a microfluidics and microarray-based immunoassay system. *Talanta* **2018**, *186*, 299–305.
7. Luo, Y.; Zhu, X.; Zhang, P.; Shen, Q.; Wang, Z.; Wen, X.; Wang, L.; Gao, J.; Dong, J.; Yang, C.; Wu, T.; Zhu, Z.; Tian, Y. The clinical performance evaluation of novel protein chips for eleven biomarkers detection and the diagnostic model study. *Int J Clin Exp Med* **2015**, *8*, 20413–20423.
8. Lee, B.S.; Lee, J.-N.; Park, J.-M.; Lee, J.-G.; Kim, S.; Cho, Y.-K.; Ko, C. A fully automated immunoassay from whole blood on a disc. *Lab Chip* **2009**, *9*, 1548–1555.
9. Chikkaveeraiah, B.V.; Mani, V.; Patel, V.; Gutkind, J.S.; Rusling, J.F. Microfluidic electrochemical immunoarray for ultrasensitive detection of two cancer biomarker proteins in serum. *Biosens Bioelectron* **2011**, *26*, 4477–4483.
10. Balakrishnan, S.R.; Hashim, U.; Gopinath, S.C.; Poopalan, P.; Ramayya, H.R.; Veeradasan, P.; Haarindraprasad, R.; Ruslinda, A.R. Polysilicon nanogap lab-on-chip facilitates multiplex analyses with single analyte. *Biosens Bioelectron* **2016**, *84*, 44–52.
11. Gao, Y.; Huo, W.; Zhang, L.; Lian, J.; Tao, W.; Song, C.; Tang, J.; Shi, S.; Gao, Y. Multiplex measurement of twelve tumor markers using a GMR multi-biomarker immunoassay biosensor. *Biosens Bioelectron* **2019**, *123*, 204–210.
